# The Absolute Isotopic Composition and Atomic Weight of Terrestrial Nickel

**DOI:** 10.6028/jres.094.035

**Published:** 1989

**Authors:** J. W. Gramlich, E. S. Beary, L. A. Machlan, I. L. Barnes

**Affiliations:** National Institute of Standards and Technology, Gaithersburg, MD 20899

**Keywords:** absolute ratio, atomic weight, isotopie abundance, mass spectrometry, nickel, SRM 986, Standard Reference Materials, terrestrial samples

## Abstract

Twenty-nine samples of high-purity nickel metals, reagent salts and minerals, collected from worldwide sources, have been examined by high-precision isotope ratio mass spectrometry for their nickel isotopic composition. These materials were compared directly with SRM 986, certified isotopie standard for nickel, using identical measurement techniques and the same instrumentation. This survey shows no statistically significant variations among the samples investigated, indicating that the certified atomic weight and associated uncertainty for SRM 986 is applicable to terrestrial nickel samples. The atomic weight calculated for SRM 986 is 58.69335±0.00015 [2]. The currently recommended IUPAC value for terrestrial nickel is 58.69±0.01.

## 1. Introduction

For several decades, the Inorganic Analytical Research Division of the National Institute of Standards and Technology has conducted a continuing program of absolute isotopie abundance and atomic weight determinations using high-precision isotope ratio mass spectrometry. Although this program has yielded extremely accurate atomic weights for reference samples, the uncertainty associated with a generally useful atomic weight is limited by the isotopie variations among materials readily available to the scientific community or by a lack of knowledge regarding such variations. This work compares the isotopie composition and atomic weight of nickel from a global population of terrestrial nickel sources, and samples that have been purified industrially into nickel metal and reagent salts, to a Standard Reference Material (SRM 986) of accurately known isotopic composition and atomic weight [1]. This reference sample is pure nickel powder, lot F-3625 obtained from Atomergic Chemicals Corporation^1^ and is available from the Office of Standard Reference Materials at NIST.

## 2. Experimental Procedure

### 2.1 Chemical Purification of the Samples

Nickel ores, such as niccolite, linnaeite, gersdorffite, and carrollite, were obtained from the U.S. National Museum. These samples were representative of ores from mines in North America, South America, Europe, Asia, and Africa (table 1). Also listed in table 1 are commercially purified nickel metal and nickel compounds from European and American sources which were procured for this study. The reagent nickel compounds required little pretreatment, while the ores required extensive chemical separations to obtain nickel essentially free of contamination, as required for this high-precision mass spectrometric technique.

Samples of nickel ore weighing 200–600 mg were treated with about 10 g of aqua regia (3:1 HCl, HNO_3_), covered and heated until the reaction subsided. Although some undissolved material remained, the samples were evaporated to dryness, and then taken up in 10 g of HCl (1+2). The samples were then filtered into 400-mL beakers and the precipitate was discarded. Ten to fifteen g of ammonium citrate solution (400 g/L) were added to each sample filtrate to complex the Fe, and the resulting mixture was diluted to 250 mL. Thirty-five g of 1% dimethylglyoxime solution in *n*-propanol were added to each beaker and heated slowly to about 70 °C while adding NH_4_OH until the pH was between 8 and 9. The samples were heated for 30 min while maintaining the temperature at 70 °C, then cooled and filtered. The precipitate was redissolved in 4 g HNO_3_ (1+3), covered and heated until all the nickel dimethylglyoxime was dissolved. Twenty-five g of water were added to the samples as a preparation for cation exchange chromatography. Five mL of AG 50×8, 100–200 mesh cation exchange resin were placed in plastic columns about 1 cm in diameter, and the resin was cleaned using 25 g of 3 *N* HNO_3_ and then H_2_O until the eluate was neutral. After the sample was loaded onto the column the impurities were eluted using 25 g of 0.3 *N* HNO_3_. The Ni was eluted with 15 g of 3 *N* HNO_3_, and evaporated to a small volume (about 1 mL). Four g of 9 *N* HCl were added as a preparation for anion exchange chromatography. Five mL of AG 1×8, 100–200 mesh anion exchange resin was added to plastic columns 1 cm in diameter, and the resin was cleaned using 9 *N* HCL, then neutralized with water. The Ni was eluted using 12–15 g of 9 *N* HCl, while impurities remained on the column. The purified Ni solution was evaporated to dryness and redissolved in 4 g of 5 *N* HCl. The dissolved sample was then diluted to a 35-mL volume and neutralized with NH_4_OH, followed by 2 mL excess NH_4_OH. This solution was then electrolyzed at 2–2.5 V using platinum gauze electrodes until the Ni color disappeared from the solution. The electrode with the Ni deposit was removed from the solution and rinsed with H_2_O, allowed to air dry, and the electrode was weighed. The Ni was removed using concentrated HNO_3_, and the electrode was air dried and reweighed. Typically, 50–150 mg of Ni metal were recovered from each ore. Sample solutions using the purified Ni were prepared in HNO_3_ (1+49), at a concentration of 1 mg Ni/g.

The four nickel metal samples and four nickel compounds were simply dissolved, converted to the nitrate, and sample solutions were prepared as 1 mg Ni/g in HNO_3_.

### 2.2 Mass Spectrometry

Isotope ratio measurements were performed on the same NIST designed mass spectrometer that was used to determine the absolute isotopic abundance and atomic weight of a reference sample of nickel (SRM 986) [1], The measurements on terrestrial nickel materials, reported in this paper, were conducted in conjunction with, but following the mass spectrometric measurement associated with the certification of SRM 986. Analyses of SRM 986 that were used to assure measurement control and to correct for isotopic fractionation in this experiment, were also used in the calculations used to certify SRM 986. The bias-corrected isotopic ratios reported in this paper are thus based on the extensive measurements made on SRM 986 both to certify the SRM and in this work.

Samples containing approximately 5 *µ*g of nickel (as NiNO_3_ in 2% HNO_3_) were loaded onto a platinum filament with a mixture of silica gel- AlCl_3_-H_3_PO_4_ as an ionization enhancement agent. Samples were analyzed by thermal ionization mass spectrometry using procedures and parameters identical to those used for the certification of SRM 986. The details of filament preparation, sample loading and the analysis procedures have been published [1].

## 3. Results and Discussion

The corrected abundance ratios for the 29 samples are given in table 2. The same correction factors as used for SRM 986 were used. In the design of the survey both mineral type and geographic origin were considered to obtain the widest possible distribution.

The isotopie abundances reported in this work indicate no significant trend or variation in the isotopic composition of nickel in terrestrial sources.

To evaluate formally the data for constancy of isotopic composition, we used an analysis of variance (ANOVA) procedure to conduct a statistical test of the hypothesis that the materials have identical isotopic ratios. The ANOVA procedure was carried out in two ways: both including and excluding the SRM 986 data obtained from the two different instruments used in the absolute abundance ratio work. This was done to be sure that any differences between the two instruments would not affect the conclusions.

For each of the four isotope ratios, the ANOVA tests showed that the variability among the measured ratios for the minerals survey samples is never significantly greater than variability of the measurements of the SRM 986 material. Thus, the conclusion of this statistical test is that there is no evidence of real variation in the isotopie ratios for the nickel samples surveyed. The ANOVA results are summarized in Table 3.

**Figure 1:**
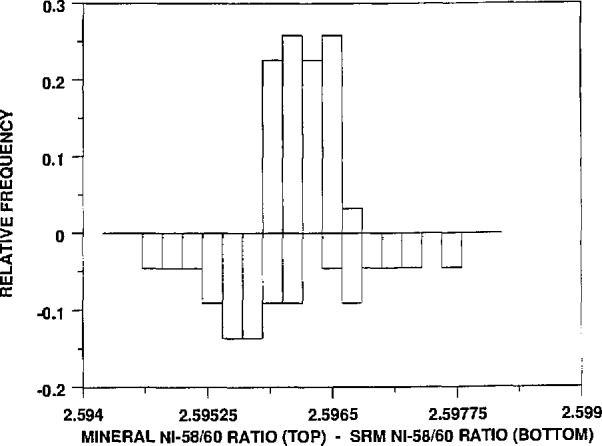
A bihistogram showing the values obtained for the ^58^Ni/^60^Ni ratios (corrected to the absolute values of the ratios obtained for SRM 986) vs those values. Shown on the top is a histogram of the mineral ^58^Ni/^60^Ni ratios and on the bottom (inverted) is a histogram of the same ratios obtained for SRM 986.

**Figure 2:**
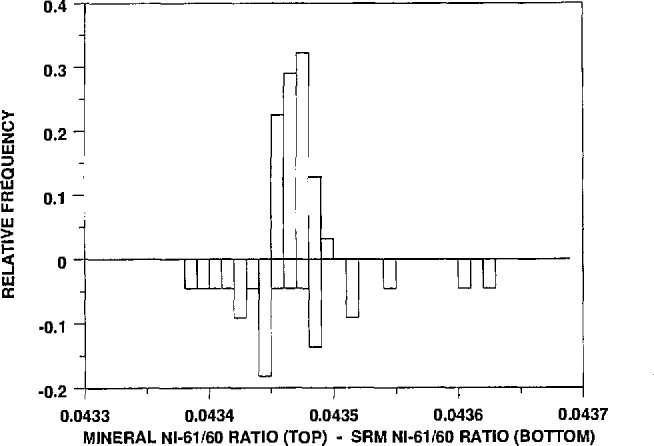
A bihistogram showing the values obtained for the ^61^Ni/^60^Ni ratios (corrected to the absolute values of the ratios obtained for SRM 986) vs those values. Shown on the top is a histogram of the mineral ^61^Ni/^60^Ni ratios and on the bottom (inverted) is a histogram of the same ratios obtained for SRM 986.

**Figure 3:**
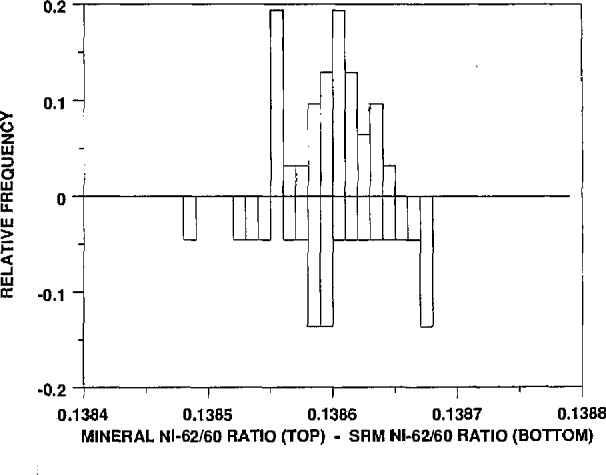
A bihistogram showing the values obtained for the ^62^Ni/^60^Ni ratios (corrected to the absolute values of the ratios obtained for SRM 986) vs those values. Shown on the top is a histogram of the mineral ^62^Ni/^60^Ni ratios and on the bottom (inverted) is a histogram of the same ratios obtained for SRM 986.

**Figure 4:**
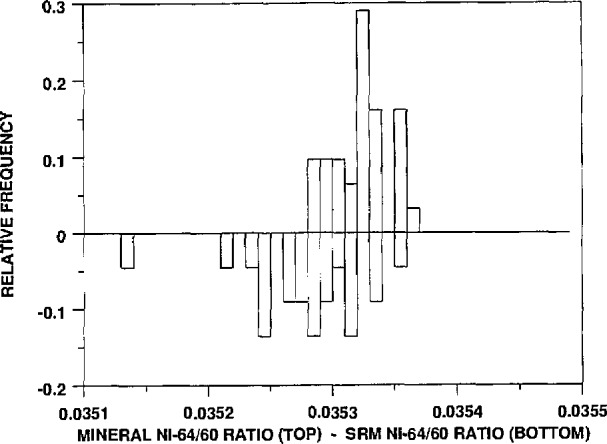
A bihistogram showing the values obtained for the ^64^Ni/^60^Ni ratios (corrected to the absolute values of the ratios obtained for SRM 986) vs those values. Shown on the top is a histogram of the mineral ^64^Ni/^60^Ni ratios and on the bottom (inverted) is a histogram of the same ratios obtained for SRM 986.

Graphical summaries of these results, in a somewhat different form, are shown in figures 1–4. These figures, called “bihistograms,” provide a visual comparison of the measured isotope ratio data for the SRM 986 material (as measured by instrument #1, see reference [1]) with the corresponding ratios for the other nickel samples surveyed. In each figure the isotope ratios for SRM 986 are represented in the “upside-down” histogram, with the histogram of the mineral survey ratios shown on top. The SRM 986 data always show at least as much variability as do the mineral survey data. This fact indicates that the observed variation in the survey nickel samples is readily accounted for by measurement variability, and therefore it supports the conclusion that there is no real variation in the isotopic composition of the nickel samples. It appears in these figures that the data for the minerals actually show less variation than that of the SRM. We beleive that this is only an indication that the procedure for the analyses was even more tightly controlled by the time that these samples were analyzed.

Evidence in the literature also indicates no identifiable variations in the atomic weight of nickel among terrestrial sources [2]. However, many of the previous measurements are far less precise than this study, and thus many have not been able to identity small isotopic variations. Further support of isotopic homogeneity is supplied by a limited study of the isotopic composition of lunar samples, directly compared to SRM 986, which also showed no significant variations relative to the SRM [3].

## Figures and Tables

**Table 1:** Nickel reagent-mineral survey (description of samples)

NIST no.	Description of sample	Source
0	NIST SRM 986 (reference sample)	
1	Linnaeite (with tetrahedraite) U.S. Natl. Museum no. B2981	Schwabengrobe (near Mussen), Germany
2	Millerite U.S. Natl. Museum no. 66501	Gap Mine, Lancaster County, PA
3	Pentlandite U.S. Natl. Museum no. 96387	Worthington Mine, Sudbury, Canada
4	Gersdorffite U.S. Natl. Museum no. 113044	Temagamy Island, Ontario, Canada
5	Gersdorffite U.S. Natl. Museum no. B3310	Merkur Mine, Ems, Germany
6	Carrollite U.S. Natl. Museum no. 122349	Kambove, Congo
7	Rammelsbergite U.S. Natl. Museum no. C693	Eisleben, Saxony, Germany
8	Gersdorffite U.S. Natl. Museum no. 120381	Ait Ahmane, Bou Azzer, Morocco
9	Millerite (with gaspeite and polydimite U.S. Natl. Museum no. 121729	Pqfuri Native Trust, Transvaal, S. Africa
10	Millerite U.S. Natl. Museum no. B1596	Victoria Mine, Littfeld, Germany
11	Millerite U.S. Natl. Museum no. 113065	Temagami, Ontario, Canada
12	Niccolite (and vaesite) U.S. Natl. Museum no. 86655	Green-Meehan Mine, Cobalt, Ontario, Canada
13	Niccolite U.S. Natl. Museum no. 105150	Alistos, Sinaloa, Mexico
14	Niccolite U.S. Natl. Museum no. 121284	Iran
15	Niccolite and Chalcanthite U.S. Natl. Museum no. 103642	Schneeberg, Saxony, Germany
16	Siegenite U.S. Natl. Museum no. B3169	Schwaben Mine, Siegerland, Germany
17	Niccolite (with rammelsbergite) U.S. Natl. Museum no. 114458	La Sorpresa Mine, Tapacari, Bolivia
18	Rammelsbergite U.S. Natl. Museum no. 117334	Mohawk #2 Mine, Keweenaw County, Michigan
19	Rammelsbergite U.S. Natl. Museum no. 94556	Hudson Bay Mine, Cobalt, Ontario, Canada
20	Heazlewoodite U.S. Natl. Museum no. 115427	Lord Brassey Mine, Heazlewood, Tasmania
21	Pentlandite in Pyrrhotite U.S. Natl. Museum no. R11297	Stare Ransko Near-Chotebor, Bohemia, Czechoslovakia
22	Linnaeite U.S. Natl. Museum no. 96797	Mineral Hill Mine, Sykesville, Maryland
23	NIST SRM 772 (magnetic moment std.) Ni metal, 99.999% pure	Leico Industries Inc., New York, NY 10019
24	Ni metal, wire	Jarrell Ash
25	Ni metal, rod lot no. 02801	Spex
26	NiO, 99.999% pure lot no. N121G0541	Atomergic Chemicals, Inc.
27	NiCl_2_-6H_2_O lot no. 634B740817	Merck
28	NiCl_2_-6H_2_O lot no. 631216/1	Union Chimique, Belge, SA
29	NiSO_4_-6H_2_O lot no. 1453	Union Chimique, Belge, SA

**Table 2:** Isotope ratios for the mineral samples corrected to SRM 986

No.	Identification	^58^Ni/^60^Ni	^61^Ni/^60^Ni	^62^Ni/^60^Ni	^64^Ni/^60^Ni
1	SI-B2981	2.596679	0.043485	0.138603	0.035355
2	SI-66501	2.596233	0.043458	0.138551	0.035327
3	SI-96387	2.596016	0.043460	0.138632	0.035327
4	SI-113044	2.596500	0.043472	0.138551	0.035359
5	SI-B3310	2.595810	0.043469	0.138649	0.035352
6	SI-122349	2.596492	0.043473	0.138555	0.035282
7	SI-C693	2.596163	0.043451	0.138559	0.035295
8	SI-120381	2.596368	0.043470	0.138571	0.035308
9	SI-121729	2.595924	0.043463	0.138598	0.035286
10	SI-B1596	2.596326	0.043469	0.138597	0.035326
11	SI-113065	2.596410	0.043493	0.138616	0.035338
12	SI-86655	2.596075	0.043468	0.138628	0.035293
13	SI-105150	2.596513	0.043467	0.138584	0.035331
14	SI-121284	2.596502	0.043477	0.138634	0.035301
15	SI-103642	2.596470	0.043469	0.138607	0.035337
16	SI-B3169	2.596537	0.043458	0.138604	0.035339
17	SI-114458	2.596135	0.043460	0.138559	0.035290
18	SI-117334	2.596225	0.043461	0.138599	0.035326
19	SI-94556	2.596113	0.043458	0.138603	0.035293
20	SI-115427	2.596205	0.043486	0.138602	0.035368
21	SI-R11297	2.596508	0.043451	0.138625	0.035358
21	SI-R11297	2.595943	0.043479	0.138570	0.035305
22	SI-96797	2.596204	0.043478	0.138595	0.035320
22	SI-96797	2.595841	0.043478	0.138556	0.035353
23	SRM 772	2.595992	0.043487	0.138606	0.035338
24	Jarrell Ash	2.595953	0.043487	0.138617	0.035329
25	Spex Ni rod	2.595996	0.043478	0.138634	0.035322
26	Atomergic	2.596071	0.043467	0.138615	0.035324
27	NiCl_2_.6H_2_O	2.596036	0.043479	0.138614	0.035320
28	NiCI_2_.6H_2_O	2.596096	0.043476	0.138589	0.035319
29	NiSO_4_.6H_2_O	2.596310	0.043480	0.138587	0.035319

**Table 3:** Results of ANOVA tests of the hypothesis of equality of isotopie ratios for SRM 986 and the other nickel samples

Isotope ratio	ANOVA *F*-ratio	Significance probability^a^
All data (including instrument #2):		
^58^Ni/^60^Ni	0.184	1.00
^61^Ni/^60^Ni	0.067	1.00
^62^Ni/^60^Ni	0.433	0.99
^64^Ni/^60^Ni	1.157	0.32
Instrument #1 data only:		
^58^Ni/^60^Ni	0.113	1.00
^61^Ni/^60^Ni	0.032	1.00
^62^Ni/^60^Ni	0.288	1.00
^64^Ni/^60^Ni	0.649	0.87

a The significance probability is the probability of obtaining an *F*-ratio as large or larger than the observed value assuming that the null hypothesis (equality of isotope ratios) is true. The results of the ANOVA test are generally considered not to show significant evidence of a difference unless the significance probability is less than 0.05.

## Footnotes

1 Certain commercial equipment, instruments, or materials are identified in this paper to specify adequately the experimental procedure. Such identification does not imply recommendation or endorsement by the National Institute of Standards and Technology, nor does it imply that the materials or equipment identified are necessarily the best available for the purpose.
